# Anti-Skin Inflammatory and Anti-Oxidative Effects of the Neoflavonoid Latifolin Isolated from *Dalbergia odorifera* in HaCaT and BJ-5ta Cells

**DOI:** 10.3390/ijms24087371

**Published:** 2023-04-17

**Authors:** Linsha Dong, Hwan Lee, Zhiming Liu, Dong-Sung Lee

**Affiliations:** College of Pharmacy, Chosun University, Dong-gu, Gwangju 61452, Republic of Korea; donglinsha011@163.com (L.D.); ghksdldi123@hanmail.net (H.L.); lzmqust@126.com (Z.L.)

**Keywords:** HaCaT, anti-skin inflammatory, BJ-5ta, anti-oxidant, latifolin

## Abstract

Skin is the first line of defense in the body against external stimulation and injury. Inflammation and oxidative stress in skin cells are the initiators and promoters of several skin diseases. Latifolin is a natural flavonoid isolated from *Dalbergia odorifera* T. Chen. This study aimed to evaluate the anti-inflammatory and antioxidant properties of latifolin. The anti-inflammatory effects were evaluated using tumor necrosis factor-α/interferon-γ (TNF-α/IFN-γ)-treated HaCaT cells, revealing that latifolin inhibited the secretion of Interleukin 6 (IL-6); Interleukin 8 (IL-8); Regulated upon Activation, Normal T Cell Expressed and Presumably Secreted (RANTES); and Macrophage-derived chemokine (MDC) while decreasing the expression of Intercellular Adhesion Molecule 1 (ICAM-1). The results of western blots and immunofluorescence demonstrated that the activation of Janus kinase 2 (JAK2), Signal transducer and activator of transcription 1 (STAT1), Signal transducer and activator of transcription 3 (STAT3), and nuclear factor kappa-light-chain-enhancer of activated B (NF-κB) cells signaling pathways were significantly inhibited by latifolin. The antioxidant properties were evaluated using *t*-BHP-induced BJ-5ta cells. Latifolin increased the viability of *t*-BHP-induced BJ-5ta cells. Additionally, fluorescent staining of reactive oxygen species (ROS) showed that the production of ROS was inhibited by latifolin. Additionally, latifolin reduced the phosphorylation of p38 and JNK. The results indicate that latifolin has potential anti-inflammatory and antioxidant properties, and may be a candidate natural compound for the treatment of skin diseases.

## 1. Introduction

The skin is the largest organ in the human body. It acts as a barrier between the internal and external environments of the human body. It protects the body from harmful stimuli such as allergens, UV radiation, microbes, and other irritants [1,2]. Inflammation is a response to skin barrier damage. At the molecular level, the inflammatory response entails a number of intricate repair pathways connected to skin differentiation, innate immune response, and skin barrier restoration [3]. When the inflammatory response initially occurs, keratinocytes, innate immune cells, dendritic cells, and inflammatory cytokines such as Interleukin-1 alpha (IL-1α), tumor necrosis factor-α (TNF-α), and Interleukin 6 (IL-6) are released by activated mast cells to cause chemokines of chemotaxis, which draw immune cells to areas of injury and infection [4]. A mild inflammatory response facilitates tissue repair and infection control. However, chemokines secreted by activated keratinocytes can further exacerbate skin tissue damage in the vicinity of the inflammatory response. The intensity and resolution of inflammation determine the severity of skin tissue damage, and regulation of inflammation is important for maintaining skin homeostasis [5,6].

Skin is the largest organ of the human body. Collagen from the connective tissue of the dermis serves as a dynamic scaffold for cell attachment, critically regulating its function, and is also a repository and regulator of effective biological mediators (growth factors, cytokines, chemokines, and mother cell proteins). In human skin, dermal fibroblasts are the main collagen-producing cells responsible for the homeostasis of dermal connective tissue [7,8]. Skin cells are often adversely affected by free radicals produced by endogenous and exogenous factors. The skin has a natural defense and regulatory function against free radicals; however, skin cells are easily affected in the presence of excess free radicals [9,10]. Reactive oxygen species (ROS), which are byproducts of cellular aerobic metabolism, include oxygen ions, peroxides, and oxygen-containing free radicals. These particles contain unpaired electrons and are highly active. Low concentrations of ROS are important for cellular signal transduction. The ROS balance is controlled by complex mechanisms. If the regulatory mechanism is unbalanced, it can lead to oxidative damage to DNA, lipids, and proteins [11]. Inducing oxidative stress and the onset of a number of diseases, excessive ROS generation can interfere with the mitochondrial membrane potential and upset the cellular redox balance [11,12,13]. ROS-mediated oxidative stress can damage collagen-rich extracellular matrix. Damage to the dermal collagen ECM can weaken the structural integrity of the skin and create abnormal tissue microenvironment, thereby promoting related skin diseases [14,15].

Medicinal plants are widely used to treat skin diseases, as their bioactive compounds contain anti-inflammatory and antioxidant properties. *Dalbergia odorifera* T. Chen is primarily distributed in the tropical regions of China. Its heartwood, “Jiang Xiang,” is used to treat thrombosis, cerebral infarction, cerebral edema, and coronary heart disease [16,17]. It has anti-inflammatory, antioxidant, antitumor, and antibacterial properties, and inhibits osteoclast differentiation and other pharmacological activities. Latifolin, a neoflavone isolated from the heartwood of *D. odorifera*, has been reported to exhibit anti-inflammatory and anticarcinogenic activities in vitro. It protects against acute myocardial ischemia induced by pituitrin and isoproterenol in rats [18]. A previous study showed that latifolin attenuates inflammatory responses by inhibiting nuclear factor-kappaB (NF-κB) activation through induction of nuclear factor-E2-related factor 2 (Nrf2)-regulated heme oxygenase-1 [19]. Latifolin promotes apoptosis cell death by inhibiting AKT pathway, and may have the potential to treat oral cell carcinoma [17]. Previous studies already showed that *D. odorifera* and its component, latifolin, have anti-inflammatory effects in in vitro and in vivo studies [18,19]. However, there is no research using latifolin on the skin which measures related activity, including anti-inflammation and antioxidation of skin. Initially, we found in the screening among the compounds from *D. odorifera* that latifolin has significant inhibitory effects on TNF-α/IFN-γ-induced inflammatory cytokines and chemokines in HaCaT cells. In addition, it could improve the survival rate of fibroblasts on the *t*-bHP-induced oxidative cell death. Therefore, we have focused and conducted a study on the mechanism of skin inflammation and protective effects of latifolin.

## 2. Results

### 2.1. Effects of Latifolin on Secretion of IL-6, IL-8, MDC, and RANTES in TNF-α/IFN-γ-Treated HaCaT Cells

**Figure 1:**
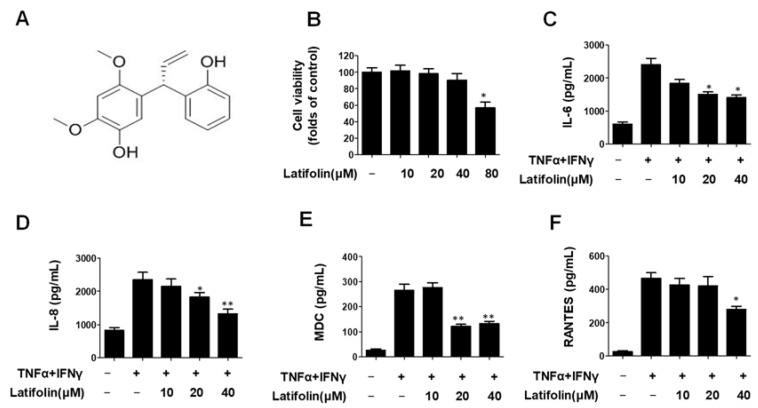
The chemical structure of latifolin (**A**) and its effects on cell viability (**B**), IL-6 (**C**), IL-8 (**D**), MDC (**E**), and RANTES (**F**) in TNF-α/IFN-γ-stimulated HaCaT cells. Cells were treated with latifolin (10–80 μM) for 24 h, and the cytotoxicity was evaluated. The cell culture supernatant was used to check ELISA kits. The data are represented as the mean ± SD (*n* = 3). * *p* < 0.05, ** *p* < 0.01 vs. TNF-α/IFN-γ-treated group.

First, we isolated latifolin from *D. odorifera* heartwood for this study, and then its structure (Figure 1A) was identified by referring to a previous study [15]. Keratinocytes are the primary epidermal cells that play a key role in the pathogenesis of skin inflammatory diseases. Keratinocytes maintain the recruitment and activation of inflammatory cells by producing various inflammatory mediators (such as cytokines and chemokines) [16]. As shown in Figure 1B–E, the secretion of Interleukin 6 (IL-6); Interleukin 8 (IL-8); Regulated upon Activation, Normal T Cell Expressed and Presumably Secreted (RANTES); and Macrophage-derived chemokine (MDC) were significantly increased by TNF-α/IFN-γ co-stimulation. Latifolin exhibited strong inhibitory effects on IL-6, IL-8, MDC, and RANTES.

### 2.2. Effects of Latifolin on TNF-α/IFN-γ-Treated ICAM-1 Expression

**Figure 2:**
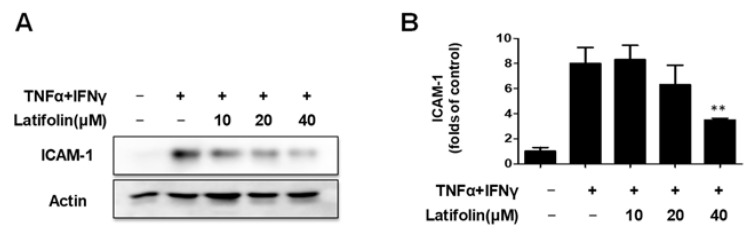
Effects of latifolin on TNF-α/IFN-γ-induced ICAM-1 expression (**A**,**B**). The data are represented as the mean ± SD (*n* = 3). ** *p* < 0.01 vs. TNF-α/IFN-γ-treated group.

Intercellular Adhesion Molecule 1 (ICAM-1) is expressed in distinct cell types, and plays an important role in cell–cell and extracellular matrix interactions, cell signaling, and immune processes [17]. ICAM-1 expression increased significantly in areas of skin inflammation. The effects of latifolin on TNF-α/IFN-γ-induced ICAM-1 expression were detected using the western blot. As shown in Figure 2A,B, latifolin downregulated ICAM-1 expression induced by TNF-α/IFN-γ-treated HaCaT cells.

### 2.3. Effects of Latifolin on JAK2/STAT1(3) Pathway in HaCaT Cells

**Figure 3:**
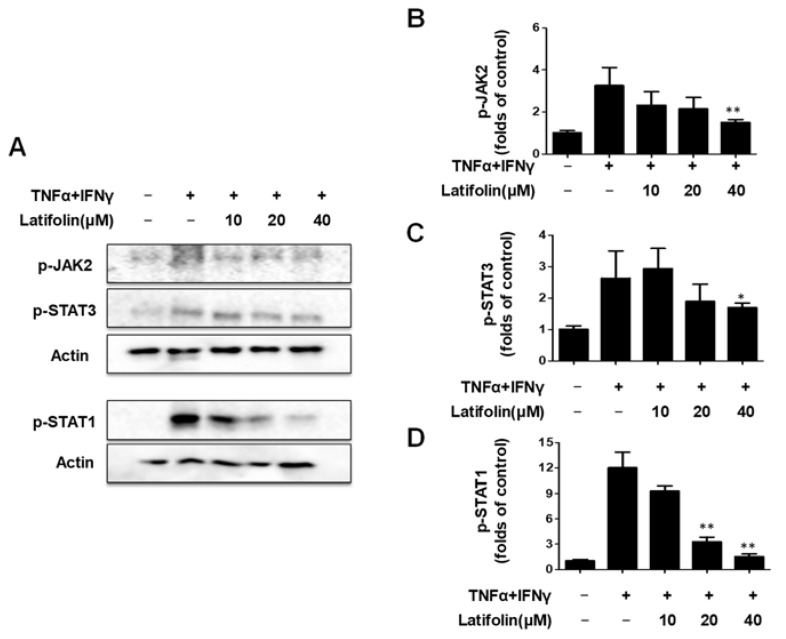
Effects of latifolin on the JAK2, STAT1, and STAT3 signaling pathways in HaCaT cells. (**A**–**D**). The data are represented as the mean ± SD (*n* = 3). * *p* < 0.05, ** *p* < 0.01 vs. TNF-α/IFN-γ-treated group.

The Janus kinase/Signal transducer and activator of transcription/pathway mediate inflammatory responses and alter the natural skin barrier, thereby stimulating the interaction between multiple cytokines to aggravate skin inflammatory symptoms [20]. As shown in Figure 3A–D, the expression of JAK2, STAT1, and STAT3 phosphorylation increased significantly after stimulation with 10 ng/mL TNF-α/IFN-γ, and latifolin significantly downregulated the phosphorylation of JAK2, STAT1, and STAT3. These findings suggested that latifolin may regulate the JAK2/STAT1/3 signaling pathway in HaCaT cells.

### 2.4. Effects of Latifolin on NF-κB Signaling Pathways in HaCaT Cells

**Figure 4:**
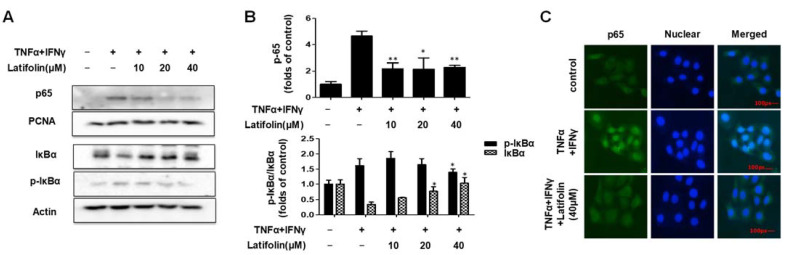
Effects of latifolin on NF-κB signaling pathways in HaCaT cells (**A**–**C**). The data are expressed as the mean ± SD (*n* = 3). * *p* < 0.05, ** *p* < 0.01 vs. TNF-α/IFN-γ-treated group.

Nuclear factor kappa-light-chain-enhancer of activated B cells (NF-κB) is a promoter of kappa light-chain synthesis. It is associated with inflammation, angiogenesis, cell proliferation, and telomerase gene expression [21,22], and is involved in the inflammatory response of the skin [23]. As shown in Figure 4A,B, the western blotting results demonstrate that the p65 translocation and p-IκBα phosphorylation induced by TNF-α/IFN-γ were inhibited by latifolin. As shown in Figure 4C, immunofluorescence results indicated that p65 translocation activity was inhibited. It revealed that latifolin has a regulatory effect on NF-κB signaling pathway; thus, it may contribute to reducing the inflammatory response.

### 2.5. Effects of Latifolin on Cell Viability in t-BHP-Induced BJ-5ta Cells

**Figure 5:**
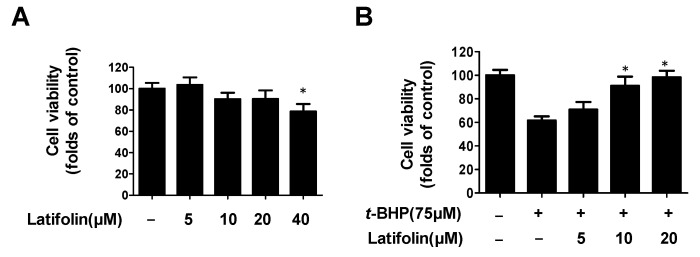
Cytotoxicity of latifolin in BJ-5ta cells (**A**). Effects of latifolin on cell viability in *t*-BHP-induced BJ-5ta cells (**B**). The data are presented as the mean ± SD (*n* = 3). * *p* < 0.05 vs. *t*-BHP group.

The results of the cytotoxicity assay of latifolin on BJ-5ta cells (shown in Figure 5A) revealed that 40 μM latifolin exhibited a certain degree of cytotoxicity. Therefore, the concentrations of latifolin utilized in the next experiments were 5, 10, and 20 μM. The effect of latifolin on *t*-BHP-induced BJ-5ta cell death was tested using a cell viability assay, and the results are demonstrated in Figure 5B. BJ-5ta cells were pretreated with latifolin (5–20 μM) for 3 h, followed by treatment with *t*-BHP (75 μM). Cell viability was reduced by *t*-BHP, and it was observed that latifolin can significantly increase cell viability in a dose-dependent manner.

### 2.6. Effects of Latifolin on ROS Production Induced by t-BHP in BJ-5ta Cells

**Figure 6:**
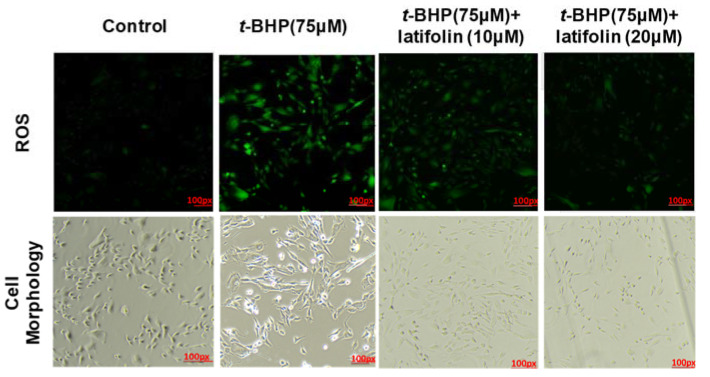
Effects of latifolin on ROS production induced by *t*-BHP in BJ-5ta cells. After pretreatment with latifolin for 3 h, ROS generation was detected in the absence or presence of *t*-BHP for 24 h using fluorescence microscope (original magnification: ×100).

Oxidative stress in dermal fibroblasts plays a crucial role in the pathogenesis of various skin diseases [24]. Antioxidants are essential for inhibition of oxidative damage and protection of skin; therefore, we measured the effect of latifolin on *t*-BHP-induced intracellular ROS accumulation using *t*-BHP in BJ-5ta cells. Fluorescence microscopy images indicated that latifolin inhibited *t*-BHP-induced ROS production in BJ-5ta cells (Figure 6); both 10 and 20 μM latifolin exhibited this ROS inhibition effect.

### 2.7. Effects of Latifolin on Mitogen-Activated Protein Kinase (MAPK) Signaling Pathways in BJ-5ta Cells

**Figure 7:**
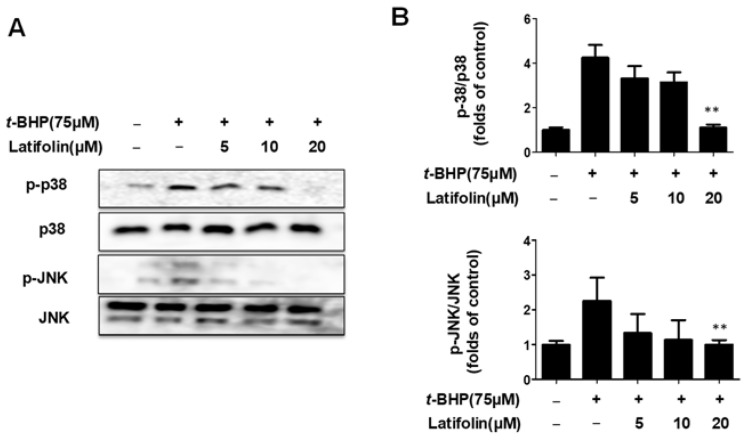
Effects of latifolin on MAPK signaling pathways in BJ-5ta cells (**A**,**B**). The data are represented as the mean ± SD (*n* = 3). ** *p* < 0.01 vs. *t*-BHP-treated group.

MAPK activation causes cell death. Studies have shown that ROS mediate MAPK phosphorylation, leading to neuronal cell death [25]. Therefore, we investigated whether latifolin inhibited MAPK activation. The *t*-BHP increased the phosphorylation of p38 and JNK, and latifolin inhibited the phosphorylation of p38 and JNK in a dose-dependent manner (Figure 7A,B). These results suggest that latifolin exhibits protective effects against *t*-BHP via the MAPK pathway (p38 and JNK).

## 3. Discussion

Skin barrier disruption and immune mechanisms may play a prominent role in the onset and support of skin diseases, such as atopic dermatitis, contact dermatitis, and psoriasis [26]. The most popular class of anti-inflammatory medications is called corticosteroids, yet a number of studies have documented substantial adverse consequences of long-term topical corticosteroid therapy, including skin shrinkage, telangiectasia, and reliance on recurrence. The lack of treatment adherence caused by these adverse effects drives a quest for novel, broadly effective topical and systemic medications that could update or augment the treatment of skin diseases [27].

Natural substances have unique advantages in treating many chronic diseases; for example, there are multiple action targets and few side effects. These have been widely used in skin care for hundreds of years, and we are currently searching for new natural substances with biological activities. These substances can promote skin health and protect the skin from harmful factors [28,29]. Reports showed that flavonoids from *D. odorifera* have good therapeutic effects on cardiovascular diseases, blood diseases, and other inflammation-related diseases [16]. The anti-skin inflammatory effects of *D. odorifera*, or its constituent compound, have not yet been studied. This study investigated the effects of latifolin isolated from *D. odorifera* on skin inflammation and oxidative stress. The skin is the largest organ in the human body, and acts as the first protective system from external threats such as noxious substances and pathogens [30]. As the most important cell type in the skin, keratinocytes play an important immune function. By secreting cytokines and chemokines, keratinocytes can recruit, activate, and regulate immune cells [30,31]. Oxidative stress can cause significant damage to the skin, reducing ECM proteins in fibroblasts, which are important for maintaining skin health. When ECM proteins are damaged, they can weaken the protective function of the skin, leading to slow wound repair and frequent skin inflammation [32].

Keratinocytes can express the receptor of TNF-α and IFN-γ. Stimulation with these cytokines can induce the expression of various proinflammatory genes in keratinocytes, such as CXCL5, CXCL8 (also known as IL-8), and intercellular adhesion molecule 1 (ICAM-1) [33]. In this study, we explored the effects of latifolin on skin inflammation and oxidative stress. When the epidermal barrier of the skin is damaged, it secretes many inflammatory factors and chemokines, such as IL-6, IL-8, IL-1β, RANTES, MDC, and Thymus and Activation Regulated Chemokine (TARC), which will further promote keratinocytes to contribute to the emergence of inflammatory skin diseases. Inhibiting the secretion of proinflammatory factors and chemokines is an effective strategy for treating inflammatory skin diseases. Latifolin demonstrated an adequate effective activity on cytokines and chemokines, such as IL-6, IL-8, MDC, and RANTES in TNF-α/IFN-γ-stimulated HaCaT cells (Figure 1).

The NF-κB pathway is a classic pro-inflammatory signaling pathway that enhances the expression of pro-inflammatory cytokines, chemokines, and adherence factors [34]. Nuclear entry and exit of NF-κB regulate inflammation response. When exposed to drugs, the nuclear translocation of p65 showed significant changes [35]. The nuclear translocation was promoted by depredating the phosphorylated IKB proteins, and p65 in the nucleus promoted IKB transcription. In our results, p65 was significantly activated in cells exposed to TNF-α/IFN-γ; the phosphorylated IKBα also showed a significant increase. Latifolin significantly inhibits the expression of p65 in the nucleus and its nuclear translocation, and increased dephosphorylation of IKBα (Figure 4). These results suggest that latifolin can significantly regulate the NF-κB pathway to exert an anti-inflammatory effect.

The JAK/STAT is a transduction pathway that is widely expressed in many cells. The JAK/STAT pathway is activated in several immunological and inflammatory disorders [36]. The signal transduction of cytokines, chemokines, and growth hormones is significantly mediated by JAK. After attaching to the cell surface JAK receptor, the ligand phosphorylates, particularly tyrosine, form residues in the receptor’s cytoplasmic tail, generating a docking site for STATs. STATs are phosphorylated after being attracted to the receptor. Once homo- or heterodimers have formed, phosphorylated STATs continue to the nucleus to stimulate gene transcription [37]. It is known that the JAK2/STAT3 pathway can be used to treat skin irritation. We examined whether latifolin prevented JAK2, STAT1, and STAT3 from becoming phosphorylated. The outcomes demonstrated that JAK2, STAT1, and STAT3 activation are inhibited by latifolin (Figure 3). These findings imply that latifolin’s anti-inflammatory actions are caused by its suppression of the JAK/STAT system.

Reactive oxygen species (ROS) are common by-products of oxidative energy metabolism, including oxygen ion and peroxide [38], and are considered to be important physiological regulators of several intracellular signaling pathways, including the MAPK pathway. Excessive ROS cause serious damage to biological molecules in cells and mitochondria, leading to inflammation and oxidative stress. Recently, studies have shown that activation of ERK enhances cell survival, while activation of JNK and p38 MAPK induces apoptosis. These reports suggest that ERK has a protective function against cellular stress, while activation of p38 MAPK/JNK leads to apoptosis-induced death [39,40]. In this study, images of *t*-BHP-induced BJ-5ta cells showed that latifolin reduced the generation of ROS (Figure 6). ERK, JNK, and p38 respond to oxidative stress, and are activated by ROS production. ERK activation controls cell proliferation; JNK and p38 respond to cell stress and contribute to inflammation and apoptosis [29]. In our results, latifolin downregulated the phosphorylation of p38 and JNK in *t*-BHP-induced BJ-5ta cells, but did not affect ERK phosphorylation (Figure 7).

## 4. Materials and Methods

### 4.1. Plant Materials and Isolation of Latifolin

In May 2019, *D. odorifera* heartwood was bought from Daehak Hanyakguk in Iksan, Korea. The College of Pharmacy at Chosun University in Korea received a voucher specimen (CUNP-2019-05-04). Two portions of 500 g of dried *D. odorifera* heartwood were extracted twice for 3 hours using hot MeOH (2 L). With n-hexane (500 mL) and CH_2_Cl_2_, the MeOH extract (84.2 g) was diluted in 60% aqueous MeOH and partitioned twice (500 mL). Chromatography was used to separate the CH_2_Cl_2_-soluble fraction (12 g) on a silica gel column (6.5 × 60 cm) using a gradient of n-hexane/EtOAc as the eluent. There were eight fractions obtained (Fr. DO-DM1–DM8). Nine fractions were obtained from the separation of the DO-DM3 (1.3 g), utilizing chromatography on a silica gel column (6.5 × 60 cm) with n-hexane/acetone (3:1) as the eluent (Fr. DO-DM31–DO-DM39). In order to fractionally separate DO-DM36 (0.7 g) using chromatography, n-hexane/acetone (3:1) was used as the eluent on a silica gel column (6.5 × 60 cm) to produce latifolin (67.5 mg). Latifolin was deposited at Chosun University’s College of Pharmacy in Korea (Republic of Korea) (No. CUNP-2019-SC-DO-1).

### 4.2. The Structure Identification of Latifolin

The NMR spectra used for the analysis of latifolin were recorded in CD_3_OD solution after dissolving, using a JEOL (Akishima, Tokyo, Japan) Eclipse 400 MHz spectrometer (400 MHz for ^1^H and 100 MHz for ^13^C). The chemical shifts were referenced to the residual solvent peaks and are summarized as follows. By comparing the chemical shift analysis results of NMR with those reported in the reference, the compound was identified to be structurally latifolin [19].

Latifolin: ^1^H-NMR (400 MHz, CD_3_OD); δ 3.69 (3H, s, -OCH_3_), 3.83 (3H, s, -OCH_3_), 4.70–5.36 (1H, m, =CH_2 trans_, -CH-CH=CH_2_, *J* = 17.0, 10.0, 6.0 and 1.6 Hz), 6.16 (1H, m, -CH=CH_2_, *J* = 17.0, 10.0 and 6.0 Hz), 6.55 (1H, s, 3-H), 6.60 (1H, s, 6-H), 6.72–6.98 (4H, m, B ring). ^13^C-NMR (100 MHz, CD_3_OD); δ 41.44 (C-H_A_), 56.73 (2-OCH_3_), 57.46 (4-OCH_3_), 99.87 (C-3), 115.28 (=CH_2_), 115.99 (C-3’), 117.52 (C-6), 120.11 (C-5’), 125.3 (C-1), 128.1 (C-6’), 130.4 (C-4’), 130.9 (C-1’), 140.9 (C-5), 141.8 (C-Hx), 147.6 (C-4), 152.1 (C-2), 156.0 (C-2’).

### 4.3. Cell Culture and Reagents

BJ-5ta cells were cultured in a DMEM and Medium199 combination, whereas HaCaT cells were grown in DMEM. Recombinant human TNF-α, IFN-γ, and ELISA kits for IL-8, IL-6, MDC, and RANTES are from Biolegend (San Diego, CA, USA). Sigma-Aldrich was used to obtain all chemical reagents (St. Louis, MO, USA). Santa Cruz Biotechnology supplied the ICAM-1, Actin, PCNA, HRP-conjugated anti-mouse, and anti-rabbit IgG antibodies (Dallas, TX, USA). Cell Signaling Technologies sold the researchers antibodies against p-IκBα, IκBα, p65, p-JAK2, p-STAT1, p-STAT3, p-p38, p38, p-JNK, and JNK (Danvers, MA, USA).

### 4.4. MTT Assay

Latifolin (10–80 μM) and latifolin (5–40 μM) were applied to HaCaT and BJ-5ta cell lines, respectively. Following a 24-h incubation period, the cells were treated for 1 h with 3-(4,5-dimethylthiazol-2-yl)-2,5-diphenyltetrazolium bromide (MTT) at a concentration of 0.5 mg/mL. The formazan that resulted was then dissolved in DMSO. Using an ELISA microplate reader, the absorbance of the dissolved formazan was measured at 540 nm (Molecular Devices, San Jose, CA, USA).

### 4.5. IL-6, IL-8, MDC, and RANTES Detection in Cell Supernatant

HaCaT cells were plated in 24-well plates, pretreated for 3 h with latifolin (10, 20 and 40 μM), and then stimulated for 24 h with TNF-α/IFN-γ (5 ng/mL each). Following the manufacturer’s instructions, an ELISA kit was used to gauge the levels of IL-6, IL-8, RANTES, and MDC in the cell supernatants in accordance with the manufacturer’s recommendations.

### 4.6. Extraction of Total, Nuclear, and Cytosolic Protein

Latifolin was used as a pretreatment on HaCaT cells for 10–40 μM. The cells were stimulated with TNF-α/IFN-γ (5 ng/mL each) for 24 h in order to analyze ICAM-1 using a western blot. The cells were stimulated with TNF-α/IFN-γ for 15 min in order to analyze the levels of p-IκBα, IκBα, p65, p-JAK2, p-STAT1, and p-STAT3. Cells were collected and lysed in RIPA buffer for total protein analysis. To undertake nuclear and cytoplasmic protein analysis, the cells were collected, and the proteins were extracted using a Nuclear Extraction Kit (Cayman Chemical, MI, USA) in accordance with the manufacturer’s instructions. BJ-5ta cells were pre-treated with latifolin (5–20 μM), and then exposed to *t*-BHP (75 μM) for 1 h before being collected and lysed using RIPA buffer. Prior to use, all proteins were kept at −80 °C.

### 4.7. Western Blot Analysis

SDS-PAGE was used to separate the obtained proteins, and the membranes were then transferred to nitrocellulose. The membrane was blocked with 5% skim milk for 60 min, treated overnight at 4 °C with the appropriate primary antibodies (1:1000 dilution), and then incubated for 1 h at room temperature (25 °C) with a secondary antibody conjugated with horseradish peroxidase (1:5000 dilution). Certain proteins were identified using an ECL solution after being cleaned with TBST. Using ImageJ software, the bands’ optical density was examined (National Institutes of Health, Rockville, MD, USA).

### 4.8. Immunofluorescence Analysis

Immunofluorescence was used to identify the translocation of NF-κB. On glass chamber slides, HaCaT cells were plated, pretreated with latifolin (40 μM) for 3 h, and then stimulated with TNF-α/IFN-γ for 15 min. Cells were permeabilized, blocked, fixed in 4% paraformaldehyde, and then treated with NF-κB antibodies and secondary antibodies that were FITC-labeled. Cells were incubated for 5 min, then mounted on glass slides with coverslips. Using a fluorescent microscope, cells were examined and captured on camera (Nikon Optical Co, Tokyo, Japan).

### 4.9. ROS Staining

BJ-5ta cells were seeded in 6-well plates overnight and treated with latifolin (20 and 40 μM) for 3 h before being stimulated with *t*-BHP (75 μM) for 1 h. The cells were washed twice with PBS and then stained with DCF-DA (FBS-free medium) for 20 min at 37 °C. The fluorescent staining was observed using a fluorescence microscope (Nikon Eclipse, Tokyo, Japan).

### 4.10. Statistical Analysis

The results are presented as mean ± standard deviation (SD). One-way analysis of variance (ANOVA) was performed using GraphPad Software (San Diego, CA, USA), and significance was tested using Duncan’s multiple comparison test. Statistical significance was set at * *p* < 0.05, ** *p* < 0.01, *** *p* < 0.001 vs. the TNF-α/IFN-γ-treated group/t-BHP-treated group.

## 5. Conclusions

In conclusion, latifolin, isolated from *D. odorifera* heartwood, has anti-inflammatory and antioxidant properties. In TNF-α/IFN-γ-stimulated HaCaT keratinocytes, latifolin could inhibit inflammatory cytokine and chemokine secretion and the expression of ICAM-1. Activation of the JAK2, STAT1, STAT3, and NF-κB signaling pathways was also inhibited by latifolin. In *t*-BHP-stimulated BJ-5ta fibroblasts, latifolin increased cell viability and inhibited ROS production. Latifolin also inhibited p38 and JNK activation. This study demonstrates that latifolin has a certain regulatory and protective effect on skin inflammation, as well as a certain regulatory effect on oxidative stress. It has the potential to become a candidate compound for the treatment of skin inflammation-related diseases, and can also be used as a cosmetic raw material.

## Data Availability

The data presented in this study are available. Data supporting the findings of this study are available upon request from the corresponding author.
